# Extensive Countrywide Field Investigation of Somatic Cell Counts and Total Bacterial Counts in Bulk-Tank Raw Milk in Sheep Flocks in Greece

**DOI:** 10.3390/foods10020268

**Published:** 2021-01-28

**Authors:** Daphne T. Lianou, Charalambia K. Michael, Natalia G. C. Vasileiou, Efthymia Petinaki, Peter J. Cripps, Katerina Tsilipounidaki, Angeliki I. Katsafadou, Antonis P. Politis, Nikos G. Kordalis, Katerina S. Ioannidi, Dimitris A. Gougoulis, Constantina Trikalinou, Denise C. Orfanou, Ilektra A. Fragkou, Elisavet Angelidou, Eleni I. Katsarou, Athina Tzora, Marzia Albenzio, Vasia S. Mavrogianni, Mariangela Caroprese, George C. Fthenakis

**Affiliations:** 1 Veterinary Faculty, University of Thessaly, 43100 Karditsa, Greece; dlianou@vet.uth.gr (D.T.L.); cmichail@vet.uth.gr (C.K.M.); agkatsaf@vet.uth.gr (A.I.K.); apolitis.vet@gmail.com (A.P.P.); nikolaoskordalis@gmail.com (N.G.K.); kate_ioan@windowslive.com (K.S.I.); dgoug@vet.uth.gr (D.A.G.); dorfanou@vet.uth.gr (D.C.O.); hfragou@vet.uth.gr (I.A.F.); eaggel@vet.uth.gr (E.A.); elekatsarou@vet.uth.gr (E.I.K.); vmavrog@vet.uth.gr (V.S.M.); 2 Faculty of Animal Science, University of Thessaly, 41110 Larissa, Greece; vasileiounat@gmail.com; 3 University Hospital of Larissa, 41110 Larissa, Greece; petinaki@uth.gr (E.P.); tsilipoukat@gmail.com (K.T.); constantinatrikalinou@gmail.com (C.T.); 4 Institute of Veterinary Science, University of Liverpool, Neston, South Wirral CH64 7TE, UK; peterjohncripps@gmail.com; 5 Faculty of Agriculture, University of Ioannina, 47100 Arta, Greece; tzora@uoi.gr; 6 Department of Sciences of Agriculture, Food and Environment (SAFE), University of Foggia, 71122 Foggia, Italy; marzia.albenzio@unifg.it (M.A.); mariangela.caroprese@unifg.it (M.C.)

**Keywords:** bulk-tank, goat, mastitis, protein content, raw milk, sheep, slime, somatic cell counts, *Staphylococcus*, subclinical mastitis

## Abstract

Objectives were to investigate somatic cell counts (SCC) and total bacterial counts (TBC) in the raw bulk-tank milk of sheep flocks in Greece, to study factors potentially influencing increased SCC and TBC in the bulk-tank milk of sheep and to evaluate possible associations of SCC and TBC with milk content. Throughout Greece, 325 dairy sheep flocks were visited for collection of milk sampling for somatic cell counting, microbiological examination and composition measurement. Geometric mean SCC were 0.488 × 10^6^ cells mL^−1^; geometric mean TBC were 398 × 10^3^ cfu mL^−1^; 228 staphylococcal isolates were recovered form 206 flocks (63.4%). Multivariable analyses revealed annual incidence risk of clinical mastitis, age of the farmer and month into lactation period (among 53 variables) to be significant for SCC > 1.0 × 10^6^ cells mL^−1^ and month into lactation period at sampling and availability of mechanical ventilators (among 58 variables) to be significant for TBC > 1500 × 10^3^ cfu mL^−1^. Negative correlation of SCC with fat, total protein and lactose and positive correlation of SCC with added water were found. With SCC > 1.0 × 10^6^ cells mL^−1^, significant reduction of protein content (2%) was observed, whilst in flocks with SCC > 1.5 × 10^6^ cells mL^−1^, significantly lower annual milk production per ewe (42.9%) was recorded.

## 1. Introduction

Estimation of the number of leucocytes (represented by the “somatic cell counts”, SCC) in milk remains the best method for confirmation of the inflammatory response of ewes with mastitis [1]. A recent detailed study of the international literature on ovine mastitis [2] has indicated that early references on SCC in ewes’ milk dealt mostly with the establishment and application of a threshold to define subclinical mastitis in that species. Nevertheless, factors that may affect SCC in the bulk-tank milk of sheep have not been studied extensively and determined clearly [3]. Moreover, bulk milk total bacterial counts (TBC) are used to assess the bacteriological quality of raw milk. It is generally considered that SCC and TBC are related and that their values may vary depending upon the same factors [4,5]. The currently prevailing EU legislation does not enforce a legal threshold for SCC in the raw milk of sheep. For TBC, the threshold is set at 1,500,000 colony-forming-units (cfu) mL^−1^ for milk to undergo thermal processing and at 500,000 cfu mL^−1^ for milk for direct consumption [6].

**Figure 1:**
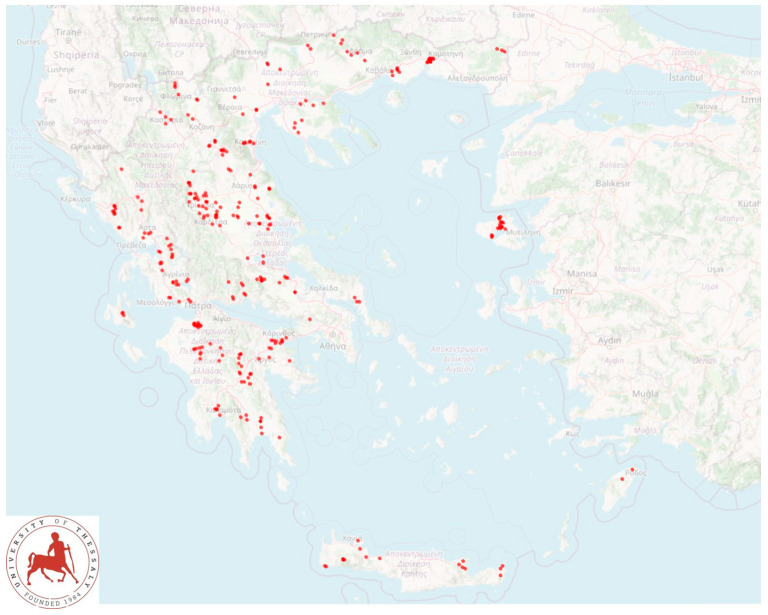
Location of 325 sheep flocks around Greece, which were visited for bulk-tank milk sampling.

The present study refers to an extensive countrywide investigation performed in 325 dairy sheep flocks throughout Greece (Figure 1). Objectives of the study were (a) to investigate SCC and TBC in the raw bulk-tank milk of sheep flocks in Greece, (b) to study factors potentially influencing increased SCC and TBC in the bulk-tank milk of sheep and (c) to evaluate possible associations of SCC and TBC with milk content.

## 2. Materials and Methods

### 2.1. Sheep Flocks and Sampling

A cross-sectional study was performed from April 2019 to July 2020. A total of 325 dairy sheep flocks in the 13 administrative regions of Greece (Figure 1) were included into the study and visited for collection of samples and information; visits had been scheduled to 327 flocks, but on two occasions (0.6%), whilst the investigators had already arrived at these farms, the respective farmers refused to collaborate. Veterinarians active in small ruminant health management around Greece, were contacted by telephone and asked if they wished to collaborate in the investigation [7]; in total, 48 veterinarians were contacted and of these, 47 (97.9%) agreed to collaborate. Flocks were selected by the collaborating veterinarians on a convenience basis (willingness of farmers to accept a visit by University personnel for an interview and sample collection). The principal investigators (D.T.L. and G.C.F.) accompanied by other investigators (C.K.M., N.G.C.V., A.P.P., N.G.K., K.S.I., D.A.G., D.C.O., E.A.) visited the respective flocks for sample collection.

At the start of each visit, an interview of the farmer was performed by using a detailed questionnaire [7] to record management practices and health issues in the flock. On each visit, four 20 mL samples were collected from the bulk-tank milk using aseptic sampling techniques (two samples were for cell counting and composition measurement and two samples were for the bacteriological examinations). Twenty-five ewes were selected at random and evaluated for body condition scoring. In order to ensure uniformity of measurements and adherence to published standards [8], scoring (0–5, including half scores) was always carried by a certified European Veterinary Specialist in Small Ruminant Health Management.

Samples were stored at 0.0 to 4.0 °C using ice packs in portable refrigerators. Somatic cell counting and milk composition measurement were performed on each of the samples within 4 h after sample collection. Transportation of samples to the laboratory was made by the investigators and by car; samples collected from flocks in the islands were also transported as ice-packed accompanying luggage by airplane (Crete, Lesvos and Rhodes) or by boat (Cephalonia).

### 2.2. Laboratory Examinations

Two of the four milk samples collected from each bulk-tank were used for somatic cell counting and milk composition measurement and the remaining two were used for the bacteriological examinations. Two sub-samples were created and processed from each of the four samples, so that each separate test was performed four times (each one in different sub-samples).

Initially, somatic cell counting (Lactoscan SCC; Milkotronic Ltd., Nova Zagora, Bulgaria) and milk composition measurement (Lactoscan Farm Eco; Milkotronic Ltd.) were performed on each of the four relevant sub-samples.

Bacteriological examinations started within 24 h after collection of samples. TBC in the milk samples were performed on each of the four relevant sub-samples. The procedures detailed by Laird et al. [9] were followed. In brief, serial 10-fold dilutions of the milk samples were made under aseptic conditions by pipetting the sample into sterile phosphate buffer saline; of each dilution, three 1 mL-drops were deposited on a Petri dish containing plate count agar (or standard methods agar); plates were incubated at 37 °C for 48 h; colony counts were performed within 2 h and based on the findings and the dilution in which growth occurred, the total bacterial count in the initial sample was calculated. Milk samples (10 μL) from each of the four relevant sub-samples were also cultured on staphylococcus selective medium (Mannitol salt agar; BioPrepare Microbiology, Athens, Greece); all plates were incubated aerobically at 37 °C for 48 h; if there was no growth, plates were re-incubated for another 24 h. After completion of sample aliquot withdrawal for microbiological examination, the temperature of the respective samples was measured and in no case was found to exceed 3.8 °C. Bacterial isolation and initial identification were performed using standard methods [10,11]. Detection of at least three confirmed staphylococcal colonies on at least one agar plate of the four plates cultured with each bulk-tank milk sub-sample from each flock, was considered to indicate presence of the organism. The staphylococcal isolates were identified to species level by using Matrix-Assisted Laser Desorption/Ionization Time-of-Flight Mass Spectrometry (VITEK MS; BioMerieux, Marcy-l’-Étoile, France). In brief, isolates were smeared from Petri dishes onto target slides and then 1 μL VITEK MS matrix was applied over the sample and air-dried and allowed to co-crystallize with the sample; target slides with all so-prepared isolates were loaded into the VITEK MS system. Then, mass spectra of whole bacterial cell proteins were acquired and compared to the known mass spectra included in the database for each staphylococcal species.

All staphylococcal isolates, were processed for evaluation of in vitro biofilm formation. This was tested by a combination of (a) culture appearance on Congo Red agar plates and (b) results of microplate adhesion test, as detailed by Vasileiou et al. [12] for staphylococcal isolates recovered from sheep milk.

### 2.3. Data Management and Analysis

#### 2.3.1. Data Management

During cell counting, total bacterial counting and milk composition measurement, for each bulk-tank milk sample, the results of the two sub-samples from each sample were averaged and then, the two means were again averaged for the final result regarding each bulk-tank milk. During body condition scoring, scores of the 25 ewes evaluated in each flock were averaged.

The results of testing for biofilm formation by the staphylococcal strains obtained by each method were assessed. Subsequently, results of the two methods (culture appearance on Congo Red agar and microplate adhesion) were combined [12] and staphylococcal strains were characterized as biofilm-forming or non-biofilm-forming.

For evaluation of the significance of increased SCC, the 1.0 × 10^6^ cells mL^−1^ threshold was used. For evaluation of the significance of increased TBC, the 1500 × 10^3^ cfu mL^−1^ threshold was used.

#### 2.3.2. Statistical Analysis

Data were entered into Microsoft Excel and analyzed using SPSS v. 21 (IBM Analytics, Armonk, NY, USA). Basic descriptive analysis was performed. Exact binomial confidence intervals (CI) were obtained. For all statistical analyses, SCC were transformed to somatic cell scores (SCS) as described by Wiggans and Shook [13] and Franzoi et al. [14]: SCS = log_2_(SCC/100) + 3, whilst TBC were transformed to log_10_ and the transformed data were used in the analyses; then, for presentation of the results, the transformed findings were back-transformed into 100 × 2^(SCS−3)^ and 10^log^ data, respectively.

In total, 53 or 58 variables (related to infrastructure, animals, production characteristics, health management and human resources in the flock) were evaluated for potential association with, respectively, SCC or TBC in the bulk-tank milk of these flocks (Table S1); these were either taken directly from the answers of the interview performed at the start of the visit or calculated based on these answers. For each of these variables, categories were created according to the answers of the farmers. Initially, SCC or TBC in the bulk-tank milk from the flocks were compared between the categories of each variable by using one-way analysis of variance.

The outcomes of “increased SCC in bulk-tank milk” (i.e., with SCC above the threshold of 1.0 × 10^6^ cells mL^−1^) and “increased TBC in bulk-tank milk” (i.e., with TBC above the threshold of 1500 × 10^3^ cfu mL^−1^) were considered. Exact binomial CI were obtained. Initially, the importance of predictors was assessed by using cross-tabulation with Pearson’s chi-square test and with simple logistic regression without random effects. Subsequently, multivariable models were created using mixed-effects logistic regression with flocks as the random effect, and initially offering to the model all variables, which achieved a significance of *p* < 0.2 in the univariable analysis and also were independent between them (n = 12 for increased SCC and n = 21 for increased TBC). Variables were removed from the initial model by backwards elimination. The *p* value of removal of a variable was assessed by the likelihood ratio test, and for those with a *p* value of >0.2 the variable with the largest probability was removed. This process was repeated until no variable could be removed with a *p* value of >0.2. The final multivariable test for increased SCC required the following variables: (a) month into the lactation period at sampling, (b) material of the floor of the barn, (c) clinical mastitis annual incidence risk, (d) age of lamb removal from their dams and (e) age of the farmer. The final multivariable test for increased TBC required the following variables: (a) month into lactation period at sampling, (b) availability of mechanical ventilators, (c) temperature in milk tank and (d) education of the farmer.

The potential association of the mean body condition score in each flock with the SCC or TBC was assessed by using analysis of correlation. SCC and TBC in flocks with mean body condition score > 2.50 were compared to those in flocks with mean body condition score ≤ 2.50 by using analysis of variance.

The potential association of the content (fat, total protein, lactose, added water) of the bulk-tank milk of these flocks with the SCC or TBC was assessed by using analysis of correlation. The potential association of annual per animal milk production during the previous lactation period with the SCC of the bulk-tank milk was also assessed by analysis of correlation. Correlations and correlation coefficients are those of Pearson.

In all analyses, statistical significance was defined at *p* ≤ 0.05.

## 3. Results

### 3.1. Somatic Cell Counts and Bacteriological Findings

**Figure 2:**
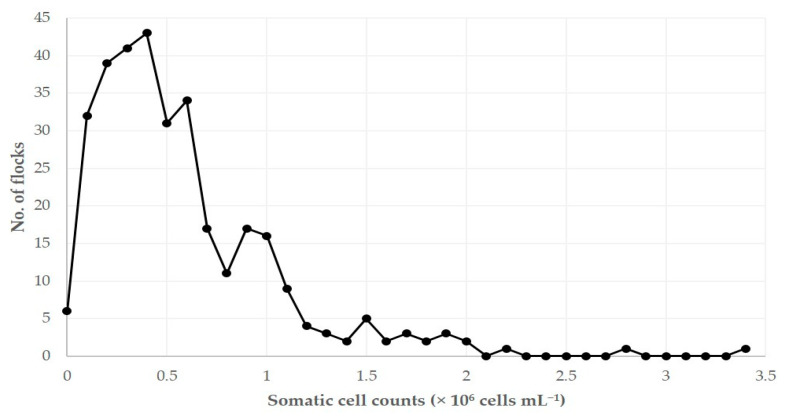
Distribution of 325 sheep flocks in Greece in accord with somatic cell counts in bulk-tank milk.

The geometric mean SCC in the bulk-tank milk of the 325 flocks visited throughout Greece and sampled was 0.488 × 10^6^ (95% CI: 0.451 × 10^6^–0.529 × 10^6^) cells mL^−1^. In 54 flocks (16.6%, 95% CI: 13.0–21.1%), SCC over 1.0 × 10^6^ cells mL^−1^ were recorded (Figure 2).

The geometric mean TBC in the bulk-tank milk of the 325 flocks was 398 × 10^3^ (95% CI: 331 × 10^3^–479 × 10^3^) cfu mL^−1^. In 58 flocks (17.9%, 95% CI: 14.1–22.4%), TBC over 1500 × 10^3^ cfu mL^−1^ were recorded. In 23 flocks (7.1%, 95% CI: 4.8–10.4%), SCC over 1.0 × 10^6^ cells mL^−1^ and TBC over 1500 × 10^3^ cfu mL^−1^ were recorded simultaneously.

Staphylococci were isolated from 206 bulk-tank milk samples (63.4%, 95% CI: 58.0–68.4%). *Staphylococcus aureus* was isolated from 54 samples (16.6%, 95% CI: 13.0–21.1%) and coagulase-negative staphylococci (cnS) from 164 samples (50.5%, 95% CI: 45.1–55.9%), resulting in total to 178 cnS isolates. Among the cnS isolates, *Staphylococcus simulans* was most often identified; other frequently identified species were *Staphylococcus equorum* and *Staphylococcus haemolyticus*. A total of 41 (75.9%) *S. aureus* and 125 (70.2%) cnS isolates were biofilm-forming. The frequency of the various coagulase-negative staphylococcal isolates identified is shown in Table 1.

**Figure 3:**
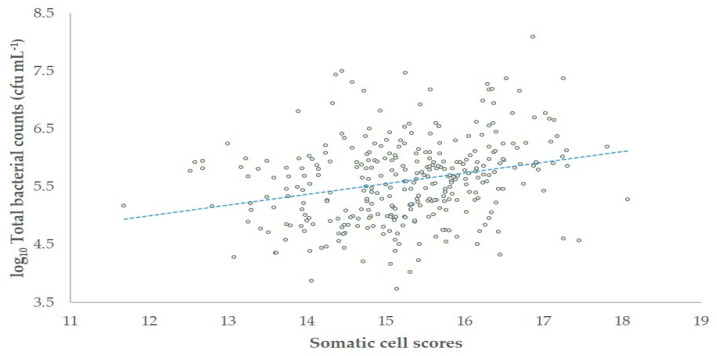
Somatic cell scores [log_2_(SCC/100) + 3] and total bacterial counts in the bulk-tank milk of 325 sheep flocks in Greece (cfu: colony-forming-units).

The correlation of SCC with TBC in the bulk-tank milk was *r* = 0.269 (*p* < 0.001) (Figure 3). There was also an association between increased SCC (>1.000 × 10^6^ cells mL^−1^) or increased TBC (>1500 × 10^3^ cfu mL^−1^) in bulk-tank milk and the isolation of *S. aureus* or biofilm-forming *S. aureus* (*p* < 0.015 in all cases), but not with the isolation of cnS (*p* > 0.35 in all cases), from that (Table 2).

There was also significant difference in the SCC between flocks, in accord with staphylococcal isolation from therein (*p* = 0.020 for all staphylococcal isolates, *p* = 0.045 for biofilm-forming staphylococcal isolates). In contrast, no such difference was seen in the TBC between flocks (*p* > 0.19 in all cases). Details are in Table 3.

### 3.2. Variables Associated with Increased Somatic Cell Counts

**Figure 4:**
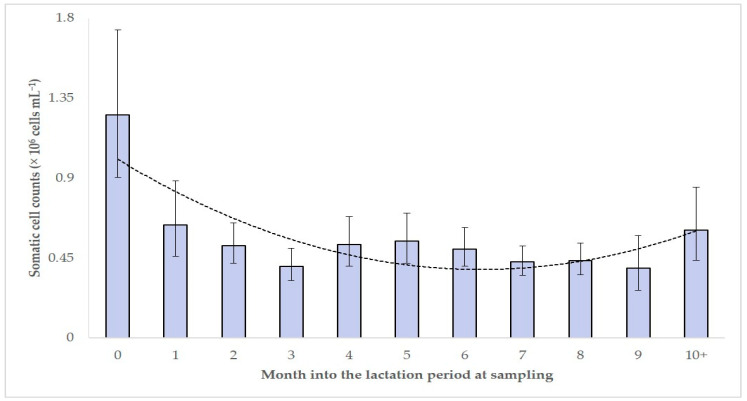
Geometric mean somatic cell counts in the bulk-tank milk of 325 sheep flocks in Greece, in accord with the month of the lactation period at which each flock was at the time of sampling (bars indicate 95% confidence intervals of the geometric mean, dotted line indicates tendency line).

**Figure 5:**
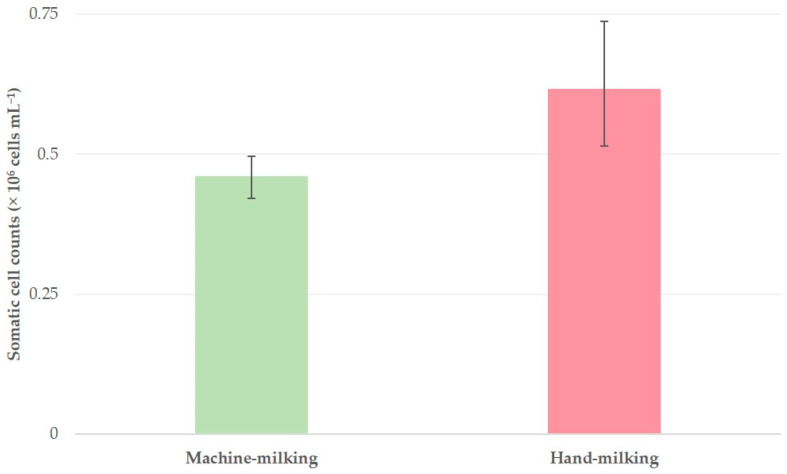
Geometric mean somatic cell counts in the bulk-tank milk of 325 sheep flocks in Greece, in accord with the milking mode practiced in the flocks (bars indicate 95% confidence intervals of the geometric mean).

For 9 of the 53 factors evaluated, the analysis indicated significant variations in SCC between their categories (Table 4, Figure 4 and Figure 5); for the other 44 no such variations were evident (Table S2).

For 4 of these 53 variables, a significant association with increased SCC (>1.0 × 10^6^ cells mL^−1^) in bulk-tank milk was evident during the univariable analysis (Table S3). Among the variables included in the multivariable analysis (Table S4), the following three emerged to be significant factors for increased SCC in the flocks: annual incidence risk of clinical mastitis in the flock (*p* = 0.001), age of the farmer (*p* = 0.006) and month into the lactation period at sampling (*p* = 0.031) (Table 5).

### 3.3. Variables Associated with Increased Total Bacterial Counts

**Figure 6:**
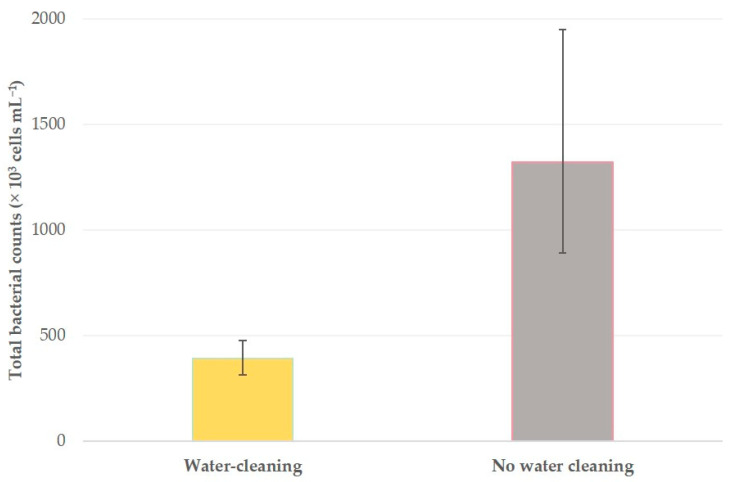
Geometric mean total bacterial counts in the bulk-tank milk of 255 sheep flocks in Greece, in accord with water cleaning of the parlor after completion of the milking procedure (bars indicate 95% confidence intervals of the geometric mean).

For 5 of the 58 factors evaluated, the analysis indicated significant variations in TBC between their categories (Table 6, Figure 6); for the other 53 no such variations were evident (Table S5).

For 12 of these 58 variables, a significant association with increased TBC (> 1500 × 10^3^ cfu mL^−1^) in bulk-tank milk was evident during the univariable analysis (Table S6). Among the variables included in the multivariable analysis (Table S7), the following two emerged to be significant factors for increased TBC in the flocks: month into the lactation period at sampling (*p* = 0.004) and availability of mechanical ventilators (*p* = 0.049) (Table 7).

### 3.4. Associations with Body Condition Score

**Figure 7:**
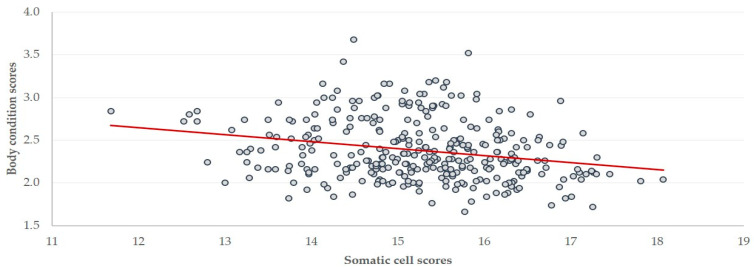
Somatic cell scores [log_2_(SCC/100) + 3] in the bulk-tank milk and mean body condition scores in 325 sheep flocks in Greece (straight line: tendency line).

There was a negative correlation of mean body condition scores with SCC (*r* = −0.240; *p* < 0.001) (Figure 7). SCC in flocks with mean body condition scores > 2.50 (n = 90) were significantly lower than SCC in flocks with mean body condition scores ≤ 2.50 (n = 235): 0.368 × 10^6^ vs. 0.544 × 10^6^ cells mL^−1^ (*p* < 0.001). It was also evident that fewer flocks with mean body condition scores > 2.50 had increased SCC (35.6%) than flocks with mean body condition scores ≤ 2.50 (56.2%) (*p* = 0.001). In contrast, there was no correlation of mean body condition scores with TBC (*r* = −0.030; *p* = 0.29).

### 3.5. Associations with Milk Composition and Milk Production

**Figure 8:**
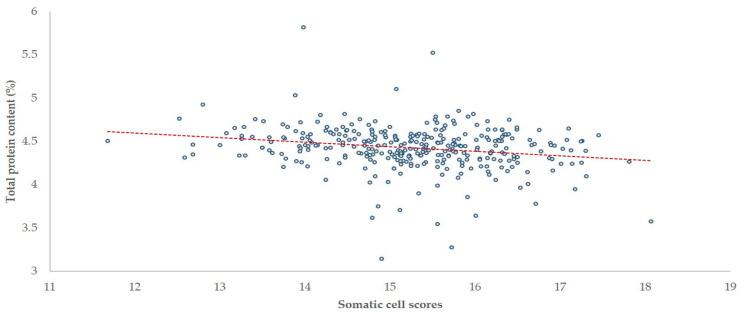
Somatic cell scores [log_2_(SCC/100) + 3] and total protein content in the bulk-tank milk of 325 sheep flocks in Greece (dashed line: tendency line).

**Figure 9:**
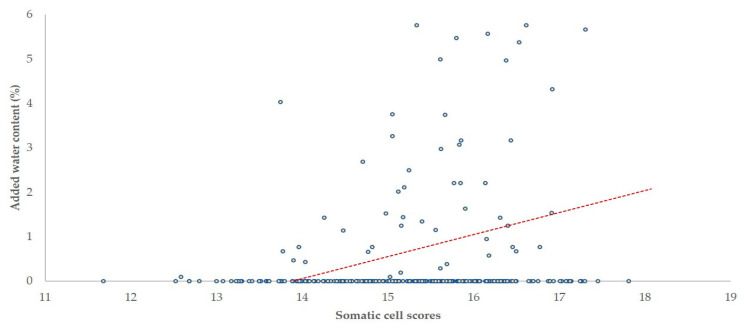
Somatic cell scores [log_2_(SCC/100) + 3] and added water content in the bulk-tank milk of 325 sheep flocks in Greece (dashed line: tendency line).

There was a negative correlation of SCC with fat, total protein and lactose content (*r* = −0.093, −0.216 and −0.171, respectively; *p* = 0.047, *p* < 0.001 and *p* = 0.001, respectively) and a positive correlation of SCC with added water content (*r* = 0.222; *p* < 0.001) in the bulk-tank milk (Figure 8 and Figure 9). No correlation was seen between TBC and content of bulk-tank milk (|*r*| < 0.085; *p* > 0.06 in all cases). There was no significant difference in the content of milk from which staphylococci or biofilm-forming staphylococci were or were not isolated (*p* > 0.065 in all cases).

Compared to flocks with SCC < 0.500 × 10^6^ cells mL^−1^, mean total protein content in the bulk-tank milk in flocks with SCC between 0.500 × 10^6^ and 1.0 × 10^6^ cells mL^−1^ was 1.0% lower (4.46% vs. 4.41%) (*p* = 0.16). Then, in flocks with SCC > 1.0 × 10^6^ cells mL^−1^, it was 2.0% lower (4.37%) (*p* = 0.022).

There was no correlation of SCC in the bulk-tank milk with annual milk production per ewe (*r* = −0.053; *p* = 0.17). Compared to flocks with SCC < 0.500 × 10^6^ cells mL^−1^, mean annual milk production per ewe in flocks with SCC > 1.500 × 10^6^ cells mL^−1^ was 42.9% lower (*p* = 0.044). 

## 4. Discussion

This paper describes an extensive field investigation in the bulk-tank milk of sheep flocks, one of largest ever on worldwide basis. Dairy sheep farming is an important sector of the agricultural industry in Greece, with a significant annual milk production. In 2019, total deliveries of sheep milk to dairy factories were 643,027,000 liters [15], accounting for approximately 20% of European and 15% of world sheep milk production [16]; this milk is used mainly for cheese production [16]. Sheep flocks from all regions of Greece were included into the study; that way, conditions prevailing throughout the country had been taken into account and factors of regional importance weighed less. In order to minimize possible bias, the study also used consistent methodologies and ensured that specific tasks were always performed by the same investigators.

Although sheep milk is of great importance for the Greek agricultural sector, no systematic countrywide investigations in the bulk-tank milk of sheep in Greece have been reported. Nationwide investigations of bulk-tank milk are important, because they allow for evaluation and monitoring of the quality of produced milk.

### 4.1. Somatic Cell Counts in Bulk-Tank Milk

In studies that appraised the relevant situation during the 1990s in the country, SCC over 1.0 × 10^6^ cells mL^−1^ in the bulk-tank milk from sheep were reported [17,18], values that are substantially higher than the ones found in the current investigation. Although it can be difficult to directly compare studies performed by differing methodological approaches, there is still merit in analyzing them, as they indicate the changes that have taken place within the last 20 to 30 years. This obvious reduction in SCC reflects the changes that have occurred in the Greek sheep industry during that period and the achievements in improving management of the flocks, benefiting from the general scientific progress in the field and the social changes in the country. The establishment of machine-milking in flocks has been an important factor that contributed in the improvement, as also corroborated in the present results (Table 3). This was coupled to the extensive import of animals of Lacaune breed (in the current study, in 106 flocks), in which sustained efforts have been made to improve low SCC [19]. The training of veterinarians active in the discipline has also improved and has led to increased implementation of udder health management practices, whilst improved training of the sheep farming community has also played a definite role (Table 3).

It is interesting to note that higher SCC values have been reported in similar studies in other countries with prominent dairy sheep farming sectors. In a study in North Spain, the mean SCC in flocks was found to be 1.072 × 10^6^ cells mL^−1^ [5], whilst in a study in Israel, the mean SCC in flocks was found to be 1.279 × 10^6^ cells mL^−1^ [20].

### 4.2. Factors Potentially Affecting Somatic Cell Counts in Bulk-Tank Milk

The present study has assessed the possible effects of a wide range of factors on SCC. This was achieved with two types of analysis: the first to identify factors that may lead to higher SCC and the second to identify factors that may lead to SCC over 1.0 × 10^6^ cells mL^−1^.

Although the current EU legislation does not mention a legal threshold for SCC in the milk of sheep, the threshold of 1.0 × 10^6^ cells mL^−1^ was considered and applied in this study, for two reasons: first, the work of Berthelot et al. [21], who indicated that in milk samples from individual ewes the value of 1.0 × 10^6^ cells mL^−1^ confirms mastitis in the animal, and second, the use of this value by some Greek dairy factories to qualitatively classify milk produced in sheep flocks and regulate prices paid to farmers. This practice is in line with a similar approach applied in Spain, where dairy factories also classify milk according to SCC [5]. Nevertheless, the bulk-milk threshold should be considered separately from the somatic cell counts in the milk of individual ewes, in which other values apply, for example Albenzio et al. [22] have indicated that an impairment of mammary gland of individual animals can be observed in values as low as 0.3 × 10^6^ cells mL^−1^.

Mastitis is the most important and significant factor associated with high SCC and the cumulative evidence from the present study confirms that SCC in the bulk-tank are mostly dependent on the presence of mastitis in a flock, with some other factors found to having some significance. In flocks with a reported annual incidence risk of clinical mastitis > 0.5%, bulk-tank milk SCC were significantly higher and also this was the most significant factor identified in the multivariable analysis for SCC over 1.0 × 10^6^ cells mL^−1^ (Table 3 and Table 4).

Other factors that proved significant for SCC over 1.0 × 10^6^ cells mL^−1^ were the month into the lactation period and the age of the farmer. The former variable was found to be particularly influential at the start of the milking period (Table 4). In previous studies [23,24], it has been found that the start of milking was a significant predisposing factor for the development of mastitis in dairy ewes; to a large extent, the present results confirm from a different viewpoint previous findings. Towards the end of a milking period, there was again an increase of SCC (Table 3), but not to the height observed at the start of the milking period. That increase has been repeatedly reported to occur in SCC of the milk of individual animals [25] and considered to occur even in the absence of infection [26]; the present results indicate that this increase was not high enough to be important.

The age of the farmers can also be of importance for increased SCC, as many variables related to management can depend upon them. A farm’s productivity has been found to decrease progressively with farmers over 45 years of age [27]. This can be explained when considering the findings of a New Zealand study, in which it was found that farmers older than 50 years were using fewer health management tools and using them less frequently than younger farmers, and this was the case even for procedures as basic as anti-clostridial vaccinations in sheep flocks [28]. This finding could be of great importance in diseases, such as mastitis, that require complex health management. It is noted that education of farmers was also important; this can also be associated with age, as younger farmers would have received some vocational or higher training, hence having better skills in flock management.

A negative correlation of SCC with mean body condition scores of ewes in the flocks was also found, a result that at first may not seem indicative of any causation. Nevertheless, one should take into account that, in sheep, the primary factor influencing body condition score is nutrition [29]. Suboptimal nutrition, which is reflected in a low body condition score, can be responsible for compromising sheep immunity through various pathways; these include the reduced formation of immunoglobulins, the inefficient cellular response, the lack of micronutrients necessary for the integrity of epithelia (e.g., zinc) or immune processes (e.g., selenium) [30], all of which play a role in the efficient defenses of sheep against mastitis pathogens [31]. Further support for this hypothesis comes from Barbagianni et al. [32], where it was shown that suboptimal nutrition throughout the final stage of pregnancy predisposed ewes to mastitis during the subsequent post-partum period.

In other relevant studies, various factors were also reported to be influencing SCC in the bulk-tank milk. For example, in the study of Gonzalo et al. [5], machine-milking, flock size, culling rate, administration of ‘dry-ewe’ treatment at the end of the lactation period, post-milking teat dipping were reported to influence SCC. In another study in Spain, only the season of sample collection (which, to some extent, is related to the month after start of the milking season) was found as a significant factor [33], whilst in a third one machine-milking and administration of ‘dry-ewe’ treatment at the end of the lactation period were found as significant factors [34]. In comparing those results to the present ones, we note that whilst some of the factors identified elsewhere were found to be of importance in our univariable models, they were not chosen by the multivariable analysis. These findings reflect the multifaceted and multifactorial nature of mastitis and the importance of the many predisposing factors [31], many of which also have complex interactions between them.

### 4.3. Bacteriological Findings and Factors Potentially Affecting Total Bacterial Counts in Bulk-Tank Milk

There was a difference between the staphylococcal species identified in this study and the species generally confirmed as aetiological agents of subclinical mastitis [35]. The present results indicate *S. simulans*, *S. equorum* and *S. haemolyticus*, as the main species identified, whilst, in general, *S. epidermidis*, *S. simulans* and *S. chromogenes* are the cnS species usually recovered from cases of subclinical mastitis. This study also recovered species that have not been considered as mastitis pathogens, e.g., *S. lugdunensis*. The above indicate that many of the staphylococci in the bulk-tank milk were not of sheep origin, but originated from other sources in the flock environment, possibly staff (*S. haemolyticus*) or other animal species (*S. intermedius*). This also indicates the possibility of contamination of the milk with bacteria of human origin, which then can act as potential human pathogens (e.g., with production of enterotoxins or transfer of antibiotic resistance genes). Albenzio et al. [36] reported that the hands of milkers were the main sources of milk contamination with bacteria of non-animal source. Although in most cases pasteurization of milk would kill such bacteria, one should also take into account the cases of cheese production made of unpasteurized milk, mainly in small-scale local cheese types.

Among the staphylococcal isolates recovered, 71.5% were identified as biofilm-forming, which indicates the high proportion of such strains even among non-sheep sources. Increased adhesion properties can lead in colonization of the milk system (teatcups, milklines, etc.), thus providing increased risk for intramammary infection of ewes at the parlor, leading to staphylococcal mastitis [14].

The difference in the sources of bacteria in milk is reflected in the difference of factors that can influence TBC to the respective factors for SCC in the bulk-tank milk. For example, water cleaning of the parlor is important (Table 5), because it reduces bacterial load in parlor equipment (e.g., milklines) and thus contributes to reducing milk contamination. Moreover, even for factors that are of importance for both high SCC and high TBC, there may be differing reasons in their significance, for example, after the start of the milking period (more frequently occurring in autumn or winter), sheep spend a lot of time indoors and animal houses are crowded (which may also occur due to presence of lambs not yet sent for slaughter): this results in increased bacterial loads within the animal houses and facilitates milk contamination and high TBC. As the lactation period advances and animal houses become less crowded, bacterial loads decrease and this is reflected in lower TBC.

The current results indicate that in most cases raw milk from sheep flocks complied with the standards required in the legislation. The value of 1500 × 10^3^ cfu mL^−1^ is in the current EU legislation the acceptable upper limit of bacterial counts in raw milk from sheep [6]. The findings indicate a significant reduction in total bacterial counts to those reported by Anyfantakis [17] and Papadopoulos [18], who indicated that TBC > 5000 × 10^3^ cfu mL^−1^ prevailed in raw milk from sheep farms in Greece during the 1990s. Again, the same reasons as for SCC would have contributed to this reduction. Sevi et al. [37] have suggested that the threshold of 0.7 × 10^6^ cells mL^−1^ for bulk-tank milk from ewes, allows for low microbial burdens in the milk and the present findings are in line with that proposal.

There are some differences between the current results and those of Gonzalo et al. [5]. In the current study, SCC were lower to those reported by Gonzalo et al. [5], whilst TBC were higher (111 × 10^3^ cfu mL^−1^ [5]). With regard to the factors potentially affecting TBC, there was a greater similarity between the two studies than for SCC, with administration of ”dry-ewe” treatment at the end of the lactation period and annual frequency of removal/clean-up of the straw bedding having been identified to be significant during the univariable analysis in both studies. The above further indicate that there is no complete association between SCC and TBC.

### 4.4. Associations with Milk Content and Milk Production

The adverse effects in the milk content found to be associated with high SCC are compatible with the effects of mastitis on milk composition of affected individual ewes [38,39]. The present study found that in cases of SCC over 1.0 × 10^6^ cells mL^−1^, the protein content in the bulk-tank milk was significantly reduced. This directly associates increased SCC in raw milk with reduced cheese production from such milk, given that protein content of milk is a primary determinant of cheese yield. In a similar approach, Sevi et al. [37] have indicated that for SCC over 0.7 × 10^6^ cells mL^−1^ the renneting ability of milk would decrease.

When milk with high SCC is delivered, dairy factories can impose penalties in the price. The correlation of high SCC with increased water content in milk suggests that farmers might try to recuperate losses in the price of milk (and amount of money received) by increasing sales volumes through addition of water in the milk.

It was also found that in flocks with SCC in bulk-tank milk > 1.5 × 10^6^ cells mL^−1^, there was a reported lower milk production per animal. This further increases the potential adverse financial effects of high SCC.

## 5. Conclusions

The results of an extensive countrywide investigation into somatic cell counts of bulk-tank milk in 325 flocks throughout Greece indicate that with regard to somatic cell counts (geometric mean: 0.488 × 10^6^ cells mL^−1^) and total bacterial counts (geometric mean: 398 × 10^3^ cfu mL^−1^), the milk can be considered of good quality, although in 17% and 18% of flocks, respectively, SCC over 1.0 × 10^6^ cells mL^−1^ and TBC over 1500 × 10^3^ cfu mL^−1^ were found. In total, 22 different *Staphylococcus* species were identified in the samples, recovered from 63% of the flocks. Some of these species are not confirmed mammary pathogens, thus indicating their potential origin in other sources in the environment of the respective flocks. The findings also underline the fact that mastitis remains the main factor influencing SCC, whilst non-infection related parameters do not appear to exert a significant influence in comparison to the infection. An adverse correlation between SCC and milk production parameters was also shown, with a significant decrease in milk production and protein content in cases of SCC > 1.5 × 10^6^ and >1.0 × 10^6^ cells mL^−1^, respectively. These findings underline the adverse financial effects of increased SCC.

## Figures and Tables

**Table 1:** Frequency of coagulase-negative staphylococcal species recovered from bulk-tank milk of 325 sheep flocks in Greece.

Staphylococcal Species	Frequency of Staphylococcal Species	
	All Isolates ^1^	Biofilm-Forming Isolates ^2^
*Staphylococcus simulans*	35 (0.197)	26 (0.743)
*Staphylococcus equorum*	23 (0.129)	16 (0.696)
*Staphylcoccus haemolyticus*	22 (0.124)	10 (0.455)
*Staphylococcus chromogenes*	13 (0.073)	8 (0.615)
*Staphylococcus lentus*	12 (0.067)	9 (0.750)
*Staphylococcus lugdunensis*	11 (0.062)	4 (0.364)
*Staphylococcus warneri*	9 (0.051)	8 (0.889)
*Staphylococcus kloosii*	7 (0.039)	7 (1.000)
*Staphylococcus capitis*	6 (0.034)	5 (0.833)
*Staphylococcus intermedius*	6 (0.034)	3 (0.500)
*Staphylococcus cohnii* subsp. *cohnii*	4 (0.022)	2 (0.500)
*Staphylococcus epidermidis*	4 (0.022)	3 (0.750)
*Staphylococcus saprophyticus*	4 (0.022)	4 (1.000)
*Staphylococcus xylosus*	4 (0.022)	3 (0.750)
*Staphylococcus auricularis*	3 (0.017)	3 (1.000)
*Staphylococcus cohnii* subsp. *urealyticum*	3 (0.017)	3 (1.000)
*Staphylococcus sciuri*	3 (0.017)	3 (1.000)
*Staphylococcus vitulinus*	3 (0.017)	3 (1.000)
*Staphylococcus hominis*	2 (0.011)	1 (0.500)
*Staphylococcus pasteuri*	2 (0.011)	2 (1.000)
*Staphylococcus carnosus*	2 (0.011)	2 (1.000)
Total	178 (1.000)	125 (0.702)

^1^ in brackets: frequency of isolation of each species; ^2^ in brackets: proportion of biofilm-forming isolates among that species.

**Table 2:** Isolation of staphylococci from bulk-tank milk of 325 sheep flocks in Greece and association with somatic cell counts or total bacterial counts in the milk.

	Flocks (n)	Frequency of Staphylococcal Isolation		
		All Isolates	*S. aureus* Isolates	Coagulase-Negative Isolates
Somatic cell counts				
All isolates				
< 1.000 × 10^6^ cells mL^−1^	271	165 (0.609)	34 (0.125) ^a^	137 (0.506)
> 1.000 × 10^6^ cells mL^−1^	54	41 (0.759)	20 (0.370) ^a^	27 (0.500)
Biofilm-forming isolates				
< 1.000 × 10^6^ cells mL^−1^	271	113 (0.417) ^b^	24 (0.086) ^c^	93 (0.343)
> 1.000 × 10^6^ cells mL^−1^	54	35 (0.648) ^b^	17 (0.345) ^c^	22 (0.407)
Total bacterial counts				
All isolates				
< 1500 × 10^3^ cfu mL^−1^	267	164 (0.614)	37 (0.139) ^d^	134 (0.502)
> 1500 × 10 cfu mL^−1^	58	42 (0.725)	17 (0.293) ^d^	30 (0.517)
Biofilm-forming isolates				
< 1500 × 10^3^ cfu^1^ mL^−1^	267	115 (0.430)	28 (0.105) ^e^	91 (0.228)
> 1500 × 10^3^ cfu mL^−1^	58	31 (0.534)	13 (0.224) ^e^	22 (0.379)

^1^ cfu: colony-forming-units. ^a–e^ *p* < 0.05 for differences between proportions with the same superscript.

**Table 3:** Mean geometric somatic cell counts and total bacterial counts in bulk-tank milk of 325 sheep flocks in Greece, in accord with staphylococcal isolation from therein.

**Somatic Cell Counts (Cells mL^−1^)**				
Results of staphylococcal isolation	All isolates		Biofilm-forming isolates	
	Flocks	SCC	Flocks	SCC
No isolation of staphylococci	124	0.418 × 10^6 a^	182	0.444 × 10^6 b^
Isolation of cnS ^1^ only	156	0.529 × 10^6 a^	111	0.551 × 10^6 b^
Isolation of *S. aureus* only	37	0.570 × 10^6 a^	28	0.578 × 10^6 b^
Isolation of cnS and *S. aureus*	8	0.575 × 10^6 a^	4	0.412 × 10^6 b^
**Total Bacterial Counts (cfu mL^−1^) ^2^**				
Results of staphylococcal isolation	All isolates		Biofilm-forming isolates	
	Flocks	TBC	Flocks	TBC
No isolation of staphylococci	124	320 × 10^3^	182	320 × 10^3^
Isolation of cnS only	156	416 × 10^3^	111	416 × 10^3^
Isolation of *S. aureus* only	37	597 × 10^3^	28	597 × 10^3^
Isolation of cnS and *S. aureus*	8	716 × 10^3^	4	716 × 10^3^

^1^ coagulase-negative staphylococci; ^2^ cfu: colony-forming-units. ^a,b^ *p* < 0.05 for differences between means with the same superscript within a column.

**Table 4:** Factors (n = 9) with significant variations between their categories with regard to somatic cell counts (geometric mean [95% confidence intervals] cells mL^−1^) in the bulk-tank milk of 325 sheep flocks in Greece.

**Month into the lactation period at sampling**								
0–1st (n = 23)	2nd–5th (n = 138)			6th–9th (n = 147)			After 9th (n = 17)	*p*
0.760 × 10^6^ (0.575 × 10^6^–1.002 × 10^6^)	0.494 × 10^6^ (0.439 × 10^6^–0.556 × 10^6^)			0.440 × 10^6^ (0.393 × 10^6^–0.490 × 10^6^)			0.608 × 10^6^ (0.436 × 10^6^–0.848 × 10^6^)	0.004
**Availability of milking parlor**								
Yes (n = 255)				No (n = 70)				*p*
0.459 × 10^6^ (0.421 × 10^6^–0.497 × 10^6^)				0.615 × 10^6^ (0.515 × 10^6^–0.738 × 10^6^)				0.002
**Availability of a waiting area before the milking parlor**								
Yes (n = 224)				No (n = 31)				*p*
0.474 × 10^6^ (0.433 × 10^6^–0.518 × 10^6^)				0.358 × 10^6^ (0.260 × 10^6^–0.461 × 10^6^)				0.034
**Years since initial establishment or most recent renovation of the milking parlor**								
Up to 10 years (n = 168)		11–20 years (n = 84)				Over 20 years (n = 3)		*p*
0.459 × 10^6^ (0.412 × 10^6^–0.508 × 10^6^)		0.441 × 10^6^ (0.385 × 10^6^–0.508 × 10^6^)				1.354 × 10^6^ (1.037 × 10^6^–1.756 × 10^6^)		0.022
**System pulsation rate to pressure ratio**								
< 3.10 (n = 45)		3.10–3.79 (n = 126)				≥ 3.80 (n = 84)		*p*
0.471 × 10^6^ (0.377 × 10^6^–0.587 × 10^6^)		0.404 × 10^6^ (0.357 × 10^6^–0.458 × 10^6^)				0.547 × 10^6^ (0.480 × 10^6^–0.625 × 10^6^)		0.008
**Month of the start of the lambing season**								
All year(n = 18)	Aug.–Sep. (n = 75)		Oct.–Nov. (n = 170)		Dec.–Jan. (n = 48)		Feb.–Jul. (n = 14)	*p*
0.554 × 10^6^ (0.427 × 10^6^–0.713 × 10^6^)	0.477 × 10^6^ (0.407 × 10^6^–0.560 × 10^6^)		0.440 × 10^6^ (0.393 × 10^6^–0.490 × 10^6^)		0.672 × 10^6^ (0.548 × 10^6^–0.819 × 10^6^)		0.566 × 10^6^ (0.407 × 10^6^–0.791 × 10^6^)	0.006
**Clinical mastitis incidence risk**								
≤ 0.50% (n = 56)				> 0.50% (n = 269)				*p*
0.411 × 10^6^ (0.357 × 10^6^–0.477 × 10^6^)				0.506 × 10^6^ (0.208 × 10^6^–1.242 × 10^6^)				0.049
**Age of lamb removal from their dams**								
< 45 days (n = 119)		45–60 days (n = 170)				> 60 days (n = 36)		*p*
0.423 × 10^6^ (0.374 × 10^6^–0.480 × 10^6^)		0.525 × 10^6^ (0.474 × 10^6^–0.583 × 10^6^)				0.558 × 10^6^ (0.424 × 10^6^–0.738 × 10^6^)		0.020
**Education of the farmer**								
Primary or secondary (n = 222)				Over secondary (n = 103)				*p*
0.519 × 10^6^ (0.474 × 10^6^–0.567 × 10^6^)				0.429 × 10^6^ (0.372 × 10^6^–0.497 × 10^6^)				0.027

**Table 5:** Results of multivariable analysis for increased somatic cell counts (> 1.0 × 10^6^ cells mL^−1^) in the bulk-tank milk of 325 sheep flocks in Greece (mixed effects logistic regression).

Variable (n = 3)	Odds Ratio ^1^ (95% Confidence Intervals)	*p*
Month into the lactation period at sampling		0.031
0 to 1st (n = 23)	5.263 (1.965–14.098)	0.001
2nd to 5th (n = 138)	1.901 (0.971–3.722)	0.061
6th to 9th (n = 147)	reference	
subsequently to 9th (n = 17)	2.047 (0.521–8.035)	0.305
Clinical mastitis annual incidence risk in the flock		0.001
< 0.5% (n = 56)	reference	
≥ 0.50% (n = 269)	6.470 (1.528–27.402)	0.011
Age of the farmer		0.006
≤ 50 years (n = 197)	reference	
> 50 years (n = 128)	2.652 (1.459–4.818)	0.001

^1^ odds ratios calculated against the lowest prevalence associations of the variables.

**Table 6:** Factors (n = 5) with significant variations between their categories with regard to total bacterial counts (geometric mean [95% confidence intervals] cfu mL^−1^) in the bulk-tank milk of 325 sheep flocks in Greece.

**Month into the lactation period at sampling**						
0–1st (n = 23)	2nd–5th (n = 138)		6th–9th (n = 147)		After 9th (n = 17)	*p*
991 × 10^3^ (427 × 10^3^–2344 × 10^3^)	419 × 10^3^ (316 × 10^3^–550 × 10^3^)		342 × 10^3^ (263 × 10^3^–437 × 10^3^)		284 × 10^3^ (162 × 10^3^–490 × 10^3^)	0.026
**Water cleaning of parlor after the milking sessions**						
Yes (n = 247)			No (n = 8)			*p*
392 × 10^3^ (316 × 10^3^–479 × 10^3^)			1323 × 10^3^ (891 × 10^3^–1950 × 10^3^)			0.038
**Average number of lambs born per ewe**						
< 1.50 (n = 280)			≥ 1.51 (n = 45)			*p*
438 × 10^3^ (363 × 10^3^–525 × 10^3^)			221 × 10^3^ (141 × 10^3^–355 × 10^3^)			0.009
**Length of animal farming experience of the farmer**						
≤ 5 years (n = 74)		6–29 years (n = 89)		≥ 30 years (n = 162)		*p*
524 × 10^3^ (363 × 10^3^–759 × 10^3^)		483 × 10^3^ (331 × 10^3^–692 × 10^3^)		315 × 10^3^ (245 × 10^3^–407 × 10^3^)		0.036
**Education of the farmer**						
Primary or secondary (n = 222)			Over secondary (n = 103)			*p*
459 × 10^3^ (363 × 10^3^–575 × 10^3^)			291 × 10^3^ (214 × 10^3^–389 × 10^3^)			0.019

**Table 7:** Results of multivariable analysis for increased bacterial counts (> 1.5 × 10^3^ cells mL^−1^) in the bulk-tank milk of 325 sheep flocks in Greece (mixed effects logistic regression).

Variable (n = 2)	Odds Ratio ^1^ (95% Confidence Intervals)	*p*
Month into the lactation period at sampling		0.004
0 to 1st (n = 23)	8.533 (0.950–76.629)	0.056
2nd to 5th (n = 138)	4.257 (0.542–33.447)	0.168
6th to 9th (n = 147)	2.520 (0.317–20.060)	0.383
subsequently to 9th (n = 17)	reference	
Availability of mechanical ventilators		0.049
Yes (n = 47)	2.540 (1.269–5.086)	0.009
No (n = 276)	reference	

^1^ odds ratios calculated against the lowest prevalence associations of the variables.

## Data Availability

Most data presented in this study are in the Supplementary Materials. The remaining data are available on request from the corresponding author. The data are not publicly available as they form part of the PhD thesis of the first author, which has not yet been examined, approved and uploaded in the official depository of PhD theses from Greek Universities.

## Supplementary Materials

The following are available online at https://www.mdpi.com/2304-8158/10/2/268/s1, Table S1: Variables evaluated for potential association with somatic cell counts (n = 53) or total bacterial counts (n = 58) in the bulk-tank milk of 325 sheep flocks in Greece, Table S2: Factors (n = 44) with no significant variations between their categories with regard to somatic cell counts (mean cells mL^−1^) in the bulk-tank milk of 325 sheep flocks in Greece, Table S3: Variables (n = 4) associated with increased somatic cell counts (> 1.0 × 10^6^ cells mL^−1^) in the bulk-tank milk of 325 sheep flocks in Greece, as found in univariable analysis, Table S4: Variables (n = 12) with *p* < 0.20 in the difference between their categories for increased somatic cell counts (i.e., > 1.0 × 10^6^ cells mL^−1^) in the bulk-tank milk of 325 sheep flocks in Greece, as found in the univariable analysis, which were then included in the multivariable analysis, Table S5: Factors (n = 53) with no significant variations between their categories with regard to total bacterial counts (geometric mean cfu mL^−1^) in the bulk-tank milk of 325 sheep flocks in Greece, Table S6: Variables (n = 12) associated with increased total bacterial counts (>1500 × 10^3^ cells mL^−1^) in the bulk-tank milk of 325 sheep flocks in Greece, as found in univariable analysis, Table S7: Variables (n = 21) with *p* < 0.20 in the between their categories for increased total bacterial counts (i.e., >1500 × 10^3^ cfu mL^−1^) in the bulk-tank milk of 325 sheep flocks in Greece, as found in the univariable analysis, which were then included in the multivariable analysis.
