# Supplementary material for: Extensive Countrywide Field Investigation of Somatic Cell Counts and Total Bacterial Counts in Bulk-Tank Raw Milk in Sheep Flocks in Greece
Source: Foods. 2021 Jan 28;10(2):268. doi: 10.3390/foods10020268 (PMC7911129; doi:10.3390/foods10020268)
Supplement: Supplementary file 1 [file foods-10-00268-s001.zip › foods-1066660-supplementary.pdf]

Extensive Countrywide Field Investigation of Somatic Cell Counts and Total Bacterial Counts in Bulk-Tank Raw Milk in Sheep Flocks in Greece

Daphne T. Lianou, Charalambia K. Michael, Natalia G.C. Vasileiou, Efthymia Petinaki, Peter J. Cripps, Katerina Tsilipounidaki, Angeliki I. Katsafadou, Antonis P. Politis, Nikos G. Kordalis, Katerina S. Ioannidi, Dimitris A. Gougoulis, Constantina Trikalinou, Denise C. Orfanou, Ilektra A. Fragkou, Elisavet Angelidou, Eleni I. Katsarou, Athina Tzora, Marzia Albenzio, Vasia S. Mavrogianni, Mariangela Caroprese and George C. Fthenakis

Table S1. Variables evaluated for potential association with somatic cell counts (n = 53) or total bacterial counts (n = 58) in the bulk-tank milk of 325 sheep flocks in Greece.

Management system applied in the flock (description according to EFSA classification)
Month into the lactation period at sampling (month)
Availability of milking parlour (yes / no)
Availability of a waiting area before the milking parlour (yes / no)
Material of the floor of the barn (cement / soil / slatted wood / slatted metal / other)
Availability of straw bedding (yes / no)
Annual frequency of removal / clean-up of the straw bedding (no. of occasions)
Availability of mechanical ventilators (yes / no)
Years since initial establishment or most recent renovation of the milking parlour (year)
Volume of the parlour (m^3)
Material of the floor of the milking parlour (cement / tile / soil / other)
Type of milking system (mobile / non-mobile)
Type of milking parlour (fishbone / circular / linear parallel / linear one-sided / other)
Number of animal positions in the parlour (no.)
Number of available milking units per animal position (no.)
Provision of feed in the milking parlour
Availability of facilities for milk yield measurement (yes / no)
Availability of milk quality indicators (yes / no)
Availability of milk flow indicators (yes / no)
System pulsation rate (p. min^{-1})
System pressure (kPa.)
System pulsation rate to pressure ratio
Type of flow line (low / high)
Frequency of check-ups of the system by farmer (no. of occasions daily)
Regular check-up of the system by technicians (yes / no)
Annual frequency of check-ups of the system by technicians (no. of occasions)
Type of system check-ups performed by technicians (dynamic / static)
Water cleaning of parlour after the milking sessions (yes / no)
Temperature of cleaning water (^oC)
Use of detergent for parlour cleaning after the milking sessions (yes / no)
Annual frequency of changing teatcups (no. of occasions)
Availability of milk tank (yes / no) [1]
Availability of mixer in the milk tank (yes / no) [1]
Temperature in milk tank (description) [1]
Frequency of milk tank cleaning (description) [1]
Frequency of milk collection (description) [1]
Grazing land available to animals (acres per animal)
No. of ewes in the flock (no.)
Breed of ewes (description)
Average age of culling ewes (years)

Month of the start of the lambing season (description)
Total milk quantity per ewe obtained during the preceding milking period (litres)
Average number of lambs born per ewe (no.)
Collaboration with a veterinarian (yes / no)
Use of laboratory diagnostic examinations in samples of milk preventively (yes / no)
Total visits made annually by veterinarians to the flock during the preceding season (no.)
Clinical mastitis annual incidence risk in the flock (%)
Nutritional modifications performed according to the reproductive stage (yes / no)
Age of lamb removal from their dams (days)
Daily number of milking sessions (no.)
Duration of the dry-period (months)
Vaccination against mastitis (yes / no)
Administration of 'dry-ewe' treatment at the end of the lactation period (yes / no)
Use of teat disinfection after milking (yes / no)
Age of the farmer (years)
Length of animal farming experience of the farmer (years)
Education of the farmer (description)
Presence of working staff in the flock (yes / no)

[1] parametres evaluated only for potential association with total bacterial counts.

Table S2. Factors (n = 44) with no significant variations between their categories with regard to somatic cell counts (mean cells mL^{-1}) in the bulk-tank milk of 325 sheep flocks in Greece.

**Management system**				
Intensive (n = 43)	Semi-intensive (n = 151)	Semi-extensive (n = 107)	Extensive (n = 24)	*p*
0.545×10^6	0.450×10^6	0.528×10^6	0.443×10^6	0.20
**Material of the floor of the barn**				
Soil (n = 292)	Other than soil (n = 33)			*p*
0.497×10^6	0.417×10^6			0.19
**Availability of straw bedding**				
Yes (n = 268)	No (n = 55)			*p*
0.488×10^6	0.491×10^6			0.96
**Annual frequency of removal / clean-up of the straw bedding**				
1–4 occasions yearly (n = 228)	> 4 occasions yearly (n = 40)			*P*
0.497×10^6	0.440×10^6			0.30
**Availability of mechanical ventilators**				
Yes (n = 47)	Yes (n = 47)			*p*
0.528×10^6	0.482×10^6			0.43
**Volume of the parlour**				
Up to 200 m^3 (n = 155)	Over 200 m^3 (n = 100)			*p*
0.441×10^6	0.487×10^6			0.27
**Material of the floor of the milking system**				
Concrete (n = 141)	Tiles (n = 50)	Soil (n = 39)	Other (n = 25)	*p*
0.452×10^6	0.523×10^6	0.404×10^6	0.469×10^6	0.36
**Type of milking system**				
Built-in system (n = 220)	Mobile system (n = 35)			*p*
0.470×10^6	0.392×10^6			0.15
**Type of milking parlour**				
Circular (n = 10)	Parallel (n = 91)	One sided (n = 152)	Other (n = 2)	*p*
0.443×10^6	0.447×10^6	0.460×10^6	1.204×10^6	0.26
**Number of animal positions in the parlour**				
Less than 24 (n = 88)	24 (n = 111)	Over 24 (n = 56)		*p*
0.443×10^6	0.443×10^6	0.520×10^6		0.31
**Number of available milking units per animal position**				
< 1 (n = 177)	1 (n = 78)			*p*
0.434×10^6	0.520×10^6			0.056
**Provision of feed in the milking parlour**				
Yes (n = 230)	No (n = 25)			*p*
0.449×10^6	0.557×10^6			0.14
**Availability of facilities for milk yield measurement**				
Yes (n = 15)	No (n = 240)			*p*
0.485×10^6	0.457×10^6			0.74
**Availability of milk quality indicators**				
Yes (n = 0)	No (n = 255)			*p*
not available	0.468×10^6			n/a
**Availability of milk flow indicators**				
Yes (n = 3)	No (n = 252)			*p*
0.468×10^6	0.458×10^6			0.96
**System pulsation rate**				
< 140 p. min^{-1} (n = 59)	140–150 p. min^{-1} (n = 147)	> 150 p. min^{-1} (n = 49)		*p*
0.403×10^6	0.474×10^6	0.485×10^6		0.27
**System pressure**				
< 38 kPa (n = 22)	38–42 kPa (n = 203)	> 42 kPa (n = 30)		*p*
0.561×10^6	0.442×10^6	0.508×10^6		0.22
**Type of flow line**				
High (n = 182)	Low (n = 55)	Other (n = 18)		*p*
0.450×10^6	0.512×10^6	0.393×10^6		0.30

**Frequency of check-ups of the system by farmer**				
Daily (n = 220)	Less frequently than daily (n = 35)			*p*
0.455×10^6	0.478×10^6			0.71
**Regular check-up of the system by technicians**				
Yes (n = 219)	No (n = 36)			*p*
0.450×10^6	0.514×10^6			0.29
**Annual frequency of check-ups of the system by technicians**				
1 check-up (n = 123)	2 check-ups (n = 54)	>2 check-ups (n = 42)		*p*
0.453×10^6	0.446×10^6	0.446×10^6		0.99
**Type of system check-ups performed by technicians**				
Full (n = 214)	Partial (n = 5)			*p*
0.447×10^6	0.587×10^6			0.37
**Water cleaning of parlour after the milking sessions**				
Yes (n = 247)	No (n = 8)			*p*
0.459×10^6	0.436×10^6			0.84
**Temperature of cleaning water**				
≤ 65 °C (n = 85)	> 65 °C (n = 170)			*p*
0.477×10^6	0.450×10^6			0.53
**Use of detergent for parlour cleaning after the milking sessions**				
Yes (n = 252)	No (n = 3)			*p*
0.460×10^6	0.374×10^6			0.61
**Annual frequency of changing teatcups**				
1 occasion (n = 130)	Less frequently than 1 occasion (n = 125)			*p*
0.448×10^6	0.470×10^6			0.58
**Grazing land available to animals**				
≤ 0.50 ac. per animal (n = 118)	0.51–2.00 ac. per animal (n = 130)	> 2.00 ac. per animal (n = 77)		*p*
0.509×10^6	0.483×10^6	0.468×10^6		0.72
**No. of ewes in the flock**				
≤ 165 ewes (n = 88)	166–330 ewes (n = 120)	331–500 ewes (n = 66)	> 500 ewes (n = 51)	*p*
0.490×10^6	0.468×10^6	0.490×10^6	0.536×10^6	0.74
**Breed of ewes**				
Greek breeds (n = 143)	Imported breeds (n = 139)	Cross-breeds (n = 43)		*p*
0.547×10^6	0.469×10^6	0.522×10^6		0.31
**Average age of culling ewes**				
≤ 6 years (n = 226)	> 6 years(n = 99)			*p*
0.475×10^6	0.522×10^6			0.27
**Total milk quantity per ewe obtained during the preceding milking period**				
≤ 200 L (n = 174)	201–400 L (n = 140)	> 400 L (n = 11)		*p*
0.478×10^6	0.503×10^6	0.474×10^6		0.81
**Average number of lambs born per ewe**				
≤ 1.50 (n = 280)	> 1.50 (n = 45)			*p*
0.503×10^6	0.411×10^6			0.080
**Collaboration with a veterinarian**				
Yes (n = 277)	No (n = 48)			*p*
0.485×10^6	0.508×10^6			0.68
**Use of laboratory diagnostic examinations in samples of milk preventively**				
Yes (n = 70)	No (n = 255)			*p*
0.488×10^6	0.488×10^6			1.00
**Total visits made annually by veterinarians to the flock during the preceding season**				
≤ 4 (n = 139)	5–7 (n = 86)	> 7 (n = 100)		*p*
0.490×10^6	0.447×10^6	0.525×10^6		0.32
**Nutritional modifications performed according to the reproductive stage**				
Yes (n = 229)	No (n = 96)			*p*
0.473×10^6	0.527×10^6			0.22
**Daily number of milking sessions**				
1 (n = 1)	2 (n = 264)	3 (n = 60)		*p*
0.730×10^6	0.478×10^6	0.534×10^6		0.29

**Duration of the dry period**			
≤ 2 months (n = 107)	> 2 months (n = 218)		*p*
0.490 × 10⁶	0.488 × 10⁶		0.94
**Vaccination against mastitis**			
Yes (n = 126)	No (n = 199)		*p*
0.480 × 10⁶	0.494×10⁶		0.73
**Administration of ‘dry-ewe’ treatment at the end of the lactation period**			
Yes (n = 53)	No (n = 272)		*p*
0.490 × 10⁶	0.488 × 10⁶		0.97
**Use of teat disinfection after milking**			
Yes (n = 52)	No (n = 273)		*p*
0.496 × 10⁶	0.487 × 10⁶		0.88
**Age of the farmer**			
Up to 50 years (n = 197)	Over 50 years (n = 128)		*p*
0.466 × 10⁶	0.518 × 10⁶		0.19
**Length of animal farming experience**			
≤ 5 years (n = 74)	6–29 years (n = 89)	> 30 years (n = 162)	*p*
0.557 × 10⁶	0.457 × 10⁶	0.477 × 10⁶	0.18
**Presence of working staff in the flock**			
Yes (n = 123)	No (n = 202)		*p*
0.517 × 10⁶	0.472 × 10⁶		0.27

Table S3. Variables (n = 4) associated with increased somatic cell counts (> 1.0 × 10^6 cells mL^{-1}) in the bulk-tank milk of 325 sheep flocks in Greece, as found in univariable analysis.

Variable	Proportion of flocks with increased SCC [1]	Odds ratio [2] (95% confidence intervals)	*p*
**Month into the lactation period at sampling**			0.006
0 to 1st (n = 23)	0.391	5.263 (1.965 – 14.098)	0.001
2nd to 5th (n = 138)	0.188	1.901 (0.971 – 3.722)	0.061
6th to 9th (n = 147)	0.109	reference	
subsequently to 9th (n = 17)	0.176	2.047 (0.521 – 8.035)	0.305
**Month of the start of the lambing season**			0.014
All year (n = 18)	0.222	2.027 (0.610 – 6.740)	0.249
August – September (n = 75)	0.147	1.220 (0.556 – 2.677)	0.621
October – November (n = 170)	0.124	reference	
December – January (n = 48)	0.333	3.548 (1.669 – 7.542)	0.001
February – July (n = 14)	0.143	1.183 (0.247 – 5.657)	0.834
**Clinical mastitis annual incidence risk in the flock**			0.001
< 0.5% (n = 56)	0.036	reference	
≥ 0.50% (n = 269)	0.193	6.470 (1.528 – 27.402)	0.011
**Age of the farmer**			0.001
≤ 50 years (n = 197)	0.112	reference	
> 50 years (n = 128)	0.250	2.652 (1.459 – 4.818)	0.001

[1] SCC: somatic cell counts, [2] odds ratios calculated against the lowest frequency of increased SCC.

Table S4. Variables (n = 12) with $p < 0.20$ in the difference between their categories for increased somatic cell counts (i.e., $> 1.0 \times 10^6$ cells mL^{-1}) in the bulk-tank milk of 325 sheep flocks in Greece, as found in the univariable analysis, which were then included in the multivariable analysis.

Variables	*p*
Month into the lactation period at sampling	0.006
Material of the floor of the barn	0.085
Annual frequency of removal / clean-up of the straw bedding	0.099
Availability of milking parlour	0.11
Average age of culling ewes	0.072
Month of the start of the lambing season	0.014
Average number of lambs born per ewe	0.13
Primary breed of ewes	0.14
Clinical mastitis annual incidence risk in the flock	0.004
Nutritional modifications performed according to the reproductive stage	0.099
Age of lamb removal from their dams	0.074
Age of the farmer	0.001

Table S5. Factors (n = 53) with no significant variations between their categories with regard to total bacterial counts (geometric mean cfu mL^{-1}) in the bulk-tank milk of 325 sheep flocks in Greece.

**Management system**				
Intensive (n = 43)	Semi-intensive (n = 151)	Semi-extensive (n = 107)	Extensive (n = 24)	*p*
387×10^3	401×10^3	414×10^3	331×10^3	0.94
**Availability of milking parlour**				
Yes (n = 255)	Yes (n = 255)			*p*
407×10^3	407×10^3			0.61
**Availability of a waiting area before the milking parlour**				
Yes (n = 224)	No (n = 31)			*p*
424×10^3	404×10^3			0.61
**Material of the floor of the barn**				
Soil (n = 292)	Other than soil (n = 33)			*p*
386×10^3	465×10^3			0.49
**Availability of straw bedding**				
Yes (n = 268)	No (n = 55)			*p*
0.488×10^6	0.491×10^6			1.00
**Annual frequency of removal / clean-up of the straw bedding**				
1–4 occasions yearly (n = 228)	> 4 occasions yearly (n = 40)			*p*
417×10^3	374×10^3			0.70
**Availability of mechanical ventilators**				
Yes (n = 47)	No (n = 276)			*p*
495×10^3	381×10^3			0.32
**Years since initial establishment or most recent renovation of the milking parlour**				
Up to 10 years (n = 168)	11–20 years (n = 84)	Over 20 years (n = 3)		*p*
420×10^3	367×10^3	1.383×10^3		0.36
**Volume of the parlour**				
Up to 200 m^3 (n = 155)	Over 200 m^3 (n = 100)			*p*
409×10^3	406×10^3			1.00
**Material of the floor of the milking system**				
Concrete (n = 141)	Tiles (n = 50)	Soil (n = 39)	Other (n = 25)	*p*
382×10^3	703×10^3	326×10^3	322×10^3	0.075
**Type of milking system**				
Built-in system (n = 220)	Mobile system (n = 35)			*p*
403×10^3	436×10^3			0.79
**Type of milking parlour**				
Circular (n = 10)	Parallel (n = 91)	One sided (n = 152)	Other (n = 2)	*p*
383×10^3	379×10^3	446×10^3	2456×10^3	0.54
**Number of animal positions in the parlour**				
Less than 24 (n = 88)	24 (n = 111)	Over 24 (n = 56)		*p*
422×10^3	379×10^3	446×10^3		0.81
**Number of available milking units per animal position**				
< 1 (n = 177)	1 (n = 78)			*p*
386×10^3	460×10^3			0.43
**Provision of feed in the milking parlour**				
Yes (n = 230)	No (n = 25)			*p*
408×10^3	398×10^3			0.14
**Availability of facilities for milk yield measurement**				
Yes (n = 15)	No (n = 240)			*p*
790×10^3	391×10^3			0.11
**Availability of milk quality indicators**				
Yes (n = 0)	No (n = 255)			*p*
not available	407×10^3			n/a
**Availability of milk flow indicators**				
Yes (n = 3)	No (n = 252)			*p*
500×10^3	406×10^3			0.82

**System pulsation rate**				
< 140 p. min^{-1} (n = 59)	140–150 p. min^{-1} (n = 147)	> 150 p. min^{-1} (n = 49)		*p*
287×10^3	497×10^3	341×10^3		0.065
**System pressure**				
< 38 kPa (n = 22)	38–42 kPa (n = 203)	> 42 kPa (n = 30)		*p*
449×10^3	374×10^3	350×10^3		0.16
**System pulsation rate to pressure ratio**				
< 3.10 (n = 46)	3.10 – 3.79 (n = 125)	≥ 3.80 (n = 84)		*p*
549×10^3	350×10^3	437×10^3		0.25
**Type of flow line**				
High (n = 182)	Low (n = 55)	Other (n = 18)		*p*
367×10^3	569×10^3	412×10^3		0.23
**Frequency of check-ups of the system by farmer**				
Daily (n = 220)	Less frequently than daily (n = 35)			*p*
404×10^3	432×10^3			0.82
**Regular check-up of the system by technicians**				
Yes (n = 219)	No (n = 36)			*p*
396×10^3	484×10^3			0.49
**Annual frequency of check-ups of the system by technicians**				
1 check-up (n = 123)	2 check-ups (n = 54)	>2 check-ups (n = 42)		*p*
397×10^3	337×10^3	556×10^3		0.34
**Type of system check-ups performed by technicians**				
Full (n = 214)	Partial (n = 5)			*p*
389×10^3	858×10^3			0.29
**Temperature of cleaning water**				
≤ 65 °C (n = 85)	> 65 °C (n = 170)			*p*
361×10^3	427×10^3			0.47
**Use of detergent for parlour cleaning after the milking sessions**				
Yes (n = 252)	No (n = 3)			*p*
409×10^3	270×10^3			0.66
**Annual frequency of changing teatcups**				
1 occasion (n = 130)	Less frequently than 1 occasion (n = 125)			*p*
405×10^3	409×10^3			1.00
**Availability of milk tank**				
Yes (n = 307)	No (n = 18)			*p*
397×10^3	404×10^3			1.00
**Availability of mixer in the milk tank**				
Yes (n = 306)	No (n = 1)			*p*
395×10^3	1800×10^3			0.36
**Temperature in milk tank**				
≤ 3.5 °C (n = 130)	> 3.5 °C (n = 177)			*p*
397×10^3	398×10^3			1.00
**Frequency of milk tank cleaning**				
Daily (n = 79)	Every two days (n = 212)	Every three days (n = 16)		*p*
441×10^3	400×10^3	268×10^3		0.55
**Frequency of milk collection**				
Daily (n = 82)	Every two days (n = 226)	Every three days (n = 17)		*p*
468×10^3	370×10^3	467×10^3		0.50
**Grazing land available to animals**				
≤ 0.50 ac. per animal (n = 118)	0.51–2.00 ac. per animal (n = 130)	> 2.00 ac. per animal (n = 77)		*p*
375×10^3	430×10^6	377×10^3		0.78
**No. of ewes in the flock**				
≤ 165 ewes (n = 88)	166–330 ewes (n = 120)	331–500 ewes (n = 66)	> 500 ewes (n = 51)	*p*
434×10^3	377×10^3	370×10^3	426×10^3	0.90
**Breed of ewes**				
Greek breeds (n = 143)	Imported breeds (n = 139)	Cross-breeds (n = 43)		*p*
445×10^3	336×10^3	472×10^3		0.28

**Average age of culling ewes**					
≤ 6 years (n = 226)	> 6 years(n = 99)				*p*
376×10^3	455×10^3				0.34
**Month of the start of the lambing season**					
All year (n = 18)	Aug. – Sep. (n = 75)	Oct. – Nov. (n = 170)	Dec. – Jan. (n = 48)	Feb. – Jul. (n = 14)	*p*
489×10^3	406×10^3	373×10^3	356×10^3	489×10^3	0.87
**Total milk quantity per ewe obtained during the preceding milking period**					
≤ 200 L (n = 174)	201–400 L (n = 140)	> 400 L (n = 11)			*p*
405×10^3	388×10^3	221×10^3			0.97
**Collaboration with a veterinarian**					
Yes (n = 277)	No (n = 48)				*p*
400×10^3	384×10^3				0.89
**Use of laboratory diagnostic examinations in samples of milk preventive**					
Yes (n = 61)	No (n = 264)				*p*
457×10^3	383×10^3				0.42
**Total visits made annually by veterinarians to the flock during the preceding season**					
≤ 4 (n = 139)	5–7 (n = 86)	> 7 (n = 100)			*p*
393×10^3	499×10^3	423×10^3			0.25
**Clinical mastitis incidence risk**					
≤ 0.50% (n = 56)	> 0.50% (n = 269)				*p*
297×10^3	423×10^3				0.14
**Nutritional modifications performed according to the reproductive stage**					
Yes (n = 229)	No (n = 96)				*p*
383×10^3	434×10^3				0.14
**Age of lamb removal from their dams**					
< 45 days (n = 119)	45 – 60 days (n = 170)	> 60 days (n = 36)			*p*
356×10^3	408×10^3	508×10^3			0.50
**Daily number of milking sessions**					
1 (n = 1)	2 (n = 264)	3 (n = 60)			*p*
370×10^3	423×10^3	373×10^3			0.37
**Duration of the dry period**					
≤ 2 months (n = 107)	> 2 months (n = 218)				*p*
453×10^3	373×10^3				0.32
**Vaccination against mastitis**					
Yes (n = 126)	No (n = 199)				*p*
463×10^3	361×10^3				0.19
**Administration of 'dry-ewe' treatment at the end of the lactation period**					
Yes (n = 53)	No (n = 272)				*p*
501×10^3	380×10^3				0.26
**Use of teat disinfection after milking**					
Yes (n = 52)	No (n = 273)				*p*
474×10^3	385×10^3				0.40
**Age of the farmer**					
Up to 50 years (n = 197)	Over 50 years (n = 128)				*P*
407×10^3	383×10^3				0.74
**Presence of working staff in the flock**					
Yes (n = 123)	No (n = 202)				*p*
410×10^3	390×10^3				0.79

Table S6. Variables (n = 12) associated with increased total bacterial counts (> 1500 × 10^3 cells mL^{-1}) in the bulk-tank milk of 325 sheep flocks in Greece, as found in univariable analysis.

Variable	Proportion of flocks with increased TBC [1]	Odds ratio [2] (95% confidence intervals)	*p*
**Management system**			0.004
Intensive (n = 43)	0.372	6.519 (1.351 – 31.461)	0.020
Semi-intensive (n = 151)	0.159	2.079 (0.458 – 9.428)	0.343
Semi-extensive (n = 107)	0.150	1.934 (0.414 – 9.040)	0.402
Extensive (n = 24)	0.083	reference	
**Month into the lactation period at sampling**			0.006
0 to 1st (n = 23)	0.348	8.533 (0.950 – 76.629)	0.056
2nd to 5th (n = 138)	0.210	4.257 (0.542 – 33.447)	0.168
6th to 9th (n = 147)	0.136	2.520 (0.3175 – 20.060)	0.383
subsequently to 9th (n = 17)	0.059	reference	
**Annual frequency of removal / clean-up of the straw bedding**			0.004
1 to 4 times yearly (n = 228)	0.215	10.676 (1.431 – 79.676)	0.021
over 4 times yearly (n = 40)	0.025	reference	
**Availability of mechanical ventilators**			0.007
Yes (n = 47)	0.319	2.540 (1.269 – 5.086)	0.009
No (n = 278)	0.156	reference	
**No. of ewes in the flock**			0.047
Up to 165 (n = 88)	0.216	3.359 (1.183 – 9.539)	0.023
166 to 330 (n = 120)	0.225	3.542 (1.293 – 9.700)	0.014
331 to 500 (n = 66)	0.076	reference	
Over 500 (n = 51)	0.137	1.941 (0.578 – 6.518)	0.283
**Average age of culling ewes**			0.006
Up to 6 years (n = 226)	0.217	2.768 (1.3029 – 5.889)	0.008
Over 6 years (n = 99)	0.091	reference	
**Month of the start of the lambing season**			< 0.001
All year (n = 18)	0.611	17.286 (4.284 – 69.740)	< 0.001
August–September (n = 75)	0.160	2.095 (0.634 – 6.924)	0.225
October–November (n = 170)	0.159	2.077 (0.689 – 6.259)	0.194
December–January (n = 48)	0.083	reference	
February–July (n = 14)	0.286	4.400 (0.937 – 20.662)	0.060
**Total visits made annually by veterinarians to the flock during the preceding season**			0.036
0–4 (n = 139)	0.237	2.519 (1.204 – 5.270)	0.014
5–7 (n = 86)	0.163	1.573 (0.673 – 3.676)	0.295
Over 7 (n = 100)	0.110	reference	
**Clinical mastitis annual incidence risk in the flock**			0.007
< 0.5% (n = 56)	0.304	2.424 (1.253 – 4.688)	0.009
≥ 0.50% (n = 269)	0.152	reference	
**Nutritional modifications performed according to the reproductive stage**			< 0.001
Yes (n = 53)	0.528	2.632 (1.236 – 5.601)	0.012
No (n = 272)	0.110	reference	

**Administration of 'dry-ewe' treatment at the end of the lactation period**			< 0.001
Yes (n = 229)	0.207	9.035 (4.672 – 17.470)	< 0.001
No (n = 96)	0.117	reference	
**Education of the farmer**			0.047
Yes (n = 229)	0.207	1.982 (1.000 – 3.927)	0.050
No (n = 96)	0.117	reference	

[1] TBC: total bacterial counts, [2] odds ratios calculated against the lowest frequency of increased SCC.

Table S7. Variables (n = 21) with $p < 0.20$ in the difference between their categories for increased total bacterial counts (i.e., > 1500 × 10^3 cfu mL^{-1}) in the bulk-tank milk of 325 sheep flocks in Greece, as found in the univariable analysis, which were then included in the multivariable analysis.

Variables	*p*
Management system	0.004
Month into the lactation period at sampling	0.031
Annual frequency of removal / clean-up of the straw bedding	0.004
Availability of mechanical ventilators	0.007
Availability of milking parlour	0.11
Temperature in milk tank	0.063
Frequency of milk tank cleaning	0.096
Grazing land available to animals	0.13
No. of ewes in the flock	0.047
Average age of culling ewes	0.006
Month of the start of the lambing season	< 0.001
Total milk quantity per ewe obtained during the preceding milking period	0.066
Primary breed of ewes	0.16
Use of laboratory diagnostic examinations in samples of milk preventively	0.11
Total visits made annually by veterinarians to the flock during the preceding season	0.036
Clinical mastitis annual incidence risk in the flock	0.007
Nutritional modifications performed according to the reproductive stage	0.010
Daily number of milking sessions	0.063
Duration of the dry-period	0.069
Administration of 'dry-ewe' treatment at the end of the lactation period	< 0.001
Education of the farmer	0.047
